# Patient and Clinician Feedback to Inform the Development of a New Pain-Specific Patient-Reported Outcome Measure for Pelvic Floor Surgery

**DOI:** 10.1007/s00192-025-06248-1

**Published:** 2025-08-01

**Authors:** Sheymonti S. Hoque, Susannah Ahern, Helen E. O’Connell, Rasa Ruseckaite

**Affiliations:** 1 School of Public Health and Preventive Medicine, Monash University, 553 St Kilda Rd, Melbourne, VIC 3004 Australia; 2 Department of Surgery, The University of Melbourne, Melbourne, VIC Australia

**Keywords:** Pelvic floor disorder, Pelvic floor procedure, Pain, Patient-reported outcome measure, Qualitative research

## Abstract

**Introduction:**

Pelvic floor procedures may result in pain, negatively affecting women’s health-related quality of life. Existing patient-reported outcome measures (PROMs) inadequately capture specific pain attributes and their relationship to pelvic floor disorders (PFDs). This study aimed to pretest items for a new pain-specific PROM post-pelvic floor surgery through focus groups/interviews.

**Methods:**

This qualitative study utilised six focus groups/interviews with 15 adult Australian and New Zealand women with PFDs experiencing post-surgical pain and mesh complications. Consolidation with the Australasian Pelvic Floor Procedure Registry Steering Committee, consisting of 11 clinicians, also occurred. Women and clinicians provided feedback regarding 35 potential items for the new pain-specific PROM. Data from the discussions were transcribed and then thematically analysed using NVivo.

**Results:**

Women and clinicians agreed the new PROM could effectively address PFDs and pelvic floor surgical pain. Their feedback guided decision-making to modify items and design the pain instrument. Women recommended removing 14 of the 35 items, and clinicians from the registry steering committee suggested removing a further five items. The preliminary PROM with 16 items has been developed under seven key pain-related domains: sensation, region, intensity and continuity, triggers, interference, comorbidities and complications, and pain relief and management.

**Conclusions:**

This qualitative study obtained direct input from women with PFDs and clinicians in formulating items for the new measure. A preliminary version of the PROM was produced from the feedback. Once fully developed and validated, the PROM could assist shared patient–clinician decision-making and track pain-related health outcomes important to women following pelvic floor surgery.

**Supplementary Information:**

The online version contains supplementary material available at 10.1007/s00192-025-06248-1.

## Introduction

Surgical interventions for pelvic floor disorders (PFDs), including stress urinary incontinence (SUI) and pelvic organ prolapse (POP) in women, may involve the use of pelvic mesh (i.e. slings, tapes, hammocks) to improve symptom outcomes and provide a stronger repair of the pelvic floor [1]. Patients opt for a pelvic floor procedure when conservative treatments such as physiotherapy and general lifestyle changes result in unsatisfactory outcomes [2]. Clinician-reported data reveal improvement in the overall outcomes in women following a procedure for SUI and POP [3]. Regardless of the benefits of the surgery, some women report experiencing pain [3, 4].

Pain related to pelvic floor procedures is complex and can range from mild discomfort to debilitating, often accompanied by other symptoms such as dyspareunia, urinary retention and bowel dysfunction [5]. Some women may also develop de novo chronic pelvic pain post-pelvic floor procedure without the insertion of mesh or mesh-related complications [4]. The risk of women developing chronic post-pelvic floor surgical pain is associated with having pre-existing pain and poor treatment or control of postoperative pain [6]. Overall, chronic pain as a symptom of post-pelvic floor surgery can lead to poor outcomes and reduced health-related quality of life (HRQoL) of women with PFDs [7].

Patient-reported outcome measures (PROMs) capture health outcomes from the perspective of patients without interpretation or adjustment from clinicians or any other individual and can be utilised to assess various health-relevant concepts such as HRQoL, functional status and patient experience [8, 9]. PROMs can be useful to measure pain as an HRQoL outcome in women with PFDs post-pelvic floor procedure, and there are available pain instruments. However, these measures have several drawbacks [10, 11].

Acceptability studies on PROMs in women post-pelvic floor procedure revealed that the women and clinicians interviewed had expressed concern over the suitability of many of the existing measures because they were too long, ambiguous or confusing [12, 13]. Furthermore, these instruments did not cover all features of PFD and pelvic floor surgery, including pain [10, 11]. A previously conducted qualitative study that explored the pain experienced by women post-pelvic floor surgery, free from clinical or other external input, identified several aspects of pain that existing measures could not address [14]. Such findings demonstrated the need for a new PROM measuring pain for women with PFDs, which could help to assess the impact of post-surgical pain [10–14].

A new pain-specific PROM for PFDs is being designed per the US Food and Drug Administration guideline for PROM development [8]. In this study, we aimed to pretest items for the new pain-specific PROM post-pelvic floor procedure through focus groups/interviews, and then produce the first draft of the instrument.

## Materials and Methods

### Design and Setting

A qualitative study involving focus groups/interviews was conducted via teleconference to pretest a list of 35 proposed items for a new pain-specific measure for PFDs post-pelvic floor procedure. Interviews occurred in cases where there was only one participant in an allocated session. Pretesting is a key stage in PROM development [15]. Utilising this method provided valuable input and feedback from participants on the wording and scoring of the proposed items for the new instrument. Each participant was involved in one focus group session.

### Proposed List of Items

We utilised a draft questionnaire with 35 items, designed previously in our mixed methods study (Supplementary Material 1). These items were generated under eight domains of a conceptual framework capturing all important aspects of pain experienced by women with PFDs post-pelvic floor surgery. The domains consisted of ‘region of pain’ (*n* = 2 items), ‘continuity of pain’ (*n* = 2 items), ‘sensation of pain’ (*n* = 3 items), ‘pain intensity’ (*n* = 3 items), ‘pain triggers’ (*n* = 4 items), ‘pain interference’ (*n* = 5 items), ‘pain relief and management’ (*n* = 7 items), and ‘comorbidities and complications’ (*n* = 9 items).

### Patient Selection and Recruitment

Study participants included adult women (≥ 18 years) from Australia and New Zealand who had undergone pelvic floor surgery for a pelvic floor disorder (POP and/or SUI) and were experiencing pain, including those whose pain may have been associated with mesh-related complications. Women who did not report pain were excluded from the study, regardless of the presence of mesh-related complications. The consumer reference group representative from the Australasian Pelvic Floor Procedure Registry (APFPR) [16] assisted with participant recruitment by distributing the study advertisement and sending out email invitations through their professional networks among consumers and in online mesh-support groups. Interested participants were emailed an explanatory statement and were asked to complete a short screening questionnaire (Table 1) to determine their eligibility. Eligible participants confirmed their participation and availability via email.

**Table 1:** Screening questions

1. What type of pelvic floor disorder have you been diagnosed with?
2. When did you have prolapse or stress incontinence surgery? Please give the year
3. Do you know with certainty that mesh was used as part of your repair? Yes/No/Unsure
4. Have you experienced any pain following your surgery?

### Data Collection

We conducted four 60-min Zoom focus groups and two interviews from 14 February to 8 May 2024. These were conducted by the first author, SSH [female, with a Bachelor in Health Sciences (Honours)]. At the time of this study, the facilitator/interviewer was a PhD candidate/researcher. The facilitator/interviewer had no relationship with any study participants, had no previous personal experience with PFDs, associated pain or other personal experiences that might bias their approach towards the topic/participants. Participants were aware of the researcher in terms of the reasons for undertaking the research. The facilitator/interviewer introduced herself at the start of the focus group and interview by name, profession and organisation. A focus group discussion guide was designed to initiate the discussions (Supplementary Material 2). We revised and refined the proposed 35 items according to the feedback and comments participants provided on the items during the discussions. The items were emailed to participants for review before the sessions. Demographic information of the participants was collected before the sessions through a demographic questionnaire (Supplementary Material 3).

### Data Analysis

Every focus group and interview session were audio recorded. The audio recording was transcribed using Microsoft Word 365 and analysed using NVivo 14 software. Data saturation was reached from the last (sixth) session, where no new concepts were identified from the data [17]. Content and thematic analyses were conducted on participant feedback received from the amended item list in the Word document and session transcriptions. The responses for each item were coded under eight themes in the item list, consisting of (1) sensation of pain, (2) region of pain, (3) pain triggers, (4) continuity of pain, (5) pain intensity, (6) pain interference, (7) pain relief and management and (8) comorbidities and complications, based on the topic guide. Participant demographic data was collated and analysed using Microsoft Excel.

### Producing the Preliminary Version of the Pain PROM

Following revision of items based on feedback from women, the items were further reviewed by the APFPR Steering Committee, consisting of 11 clinicians (gynaecologists, urogynaecologists, urologists). All clinicians provided feedback during the Steering Committee meeting, resulting in the rewording, deletion, and remerging of items, then leading to the production of the preliminary version of the instrument.

## Results

### Sample Characteristics

Fifteen adult Australian and New Zealand women (61.8 ± 8.3 years) participated in the study (Table 2). Six focus group sessions were conducted: the first session included two participants, the second and fourth sessions each had four, and the third session included three participants. The fifth and sixth sessions had one participant, so they were treated as individual interviews (Table 2). Some of the groups were smaller due to participant unavailability for the scheduled sessions, voluntary dropout, and the need to reallocate participants because of scheduling conflicts.

**Table 2:** Demographic characteristics of focus group participants

ID	Focus group session	Age (years)	Type of PFD	Surgery year(s)	Type of surgery
P1	Session 1	58	SUI & POP	2012, 2019, 2022, 2023	Enterocele, rectocele and cystocele surgery (all three procedures utilised mesh)
P2	Session 1	58	SUI & POP	1998, 2000, 2006	POP and SUI surgery Apogee, Perigee, Monarc
P3	Session 2	69	SUI & POP	2006	Sacrocolpopexy & Burch Colposuspension
P4	Session 2	57	SUI & POP	2013	Tissue Fixation System/POP mesh
P5	Session 2	63	SUI	2011	Mini Arc
P6	Session 2	56	SUI & POP	2016	Bladder reconstruction, pelvic hysterectomy and mesh
P7	Session 3	59	SUI	2016	Mesh sling
P8	Session 3	75	POP	2013	Laparoscopic mesh, Sacrohysteropexy
P9	Session 3	57	SUI & POP	2017	Partial hysterectomy, post vaginal wall repair (rectocele), episiotomy repair, Sacrocolpopexy (pelvic mesh sling), TVT (bladder mesh sling)
P10	Session 4	76	SUI & POP	2009	TVT
P11	Session 4	74	SUI & POP	2010	Mesh surgery (mesh: Gynecare PROSIMA Pelvic Floor Repair System)
P12*	Session 4	59	SUI	Not known	Not known
P13	Session 4	57	POP	2006	Implantation of a Prolift total mesh
P14	Session 5 – individual interview	45	SUI	2020, 2021, 2022, 2023	Prolapse repair, tans-obturator tape sling, bulking, mesh removal
P15	Session 6 – individual interview	62	POP	2023	Pelvic floor repair

*POP* Pelvic organ prolapse

*SUI* Stress urinary incontinence

*TVT* Tension-free vaginal tape

*P12 underwent a pelvic floor procedure, but was unable to recall the year and specific type of procedure

Eight (53.3%) women reported having both SUI and POP, four (26.7%) had SUI, and three (20.0%) had POP (Table 2). Most women (*n* = 13, 86.7%) were aware that pelvic mesh was implanted during their procedure. The types of procedures women underwent varied across the cohort. Nine women (60.0%) were married, two (13.3%) were single and one (6.7%) was in a de facto relationship. Education-wise, most of the participants held a postgraduate diploma or equivalent (*n* = 6, 40%). Just over one-quarter of the participants held a bachelor’s degree (*n* = 4, 26.7%), and three (20%) had a postgraduate degree. Only two women (13.3%) completed school at year 11.

### Item Refinement

**Fig. 1:**
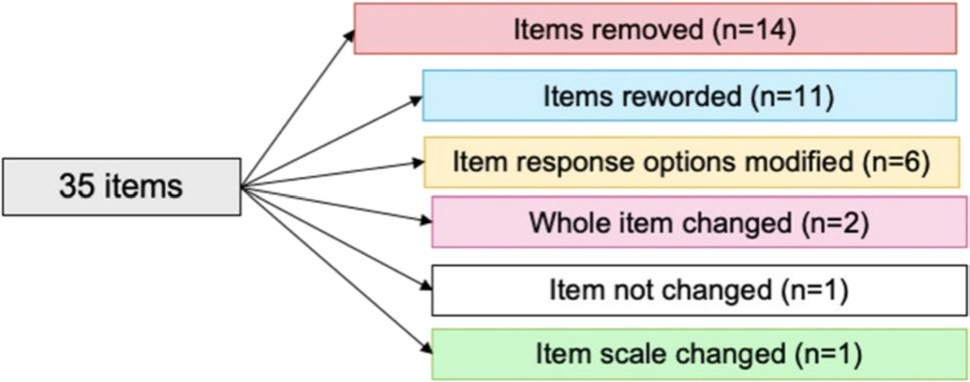
Item refinement diagram

Feedback from women helped identify problems with item content (i.e. ambiguity), response options, scale, layout and administration, as well as generate new items. As shown in Fig. 1, out of the 35 items, women recommended removing 14 redundant or irrelevant items, rewording 11 items for clarity, modifying six-item response options, replacing two items with new ones, and changing the scale of one item. Only one item was not changed. Detailed feedback for each item is provided in Table 3, and a summary of the items for each of the domains is discussed below.

**Table 3:**
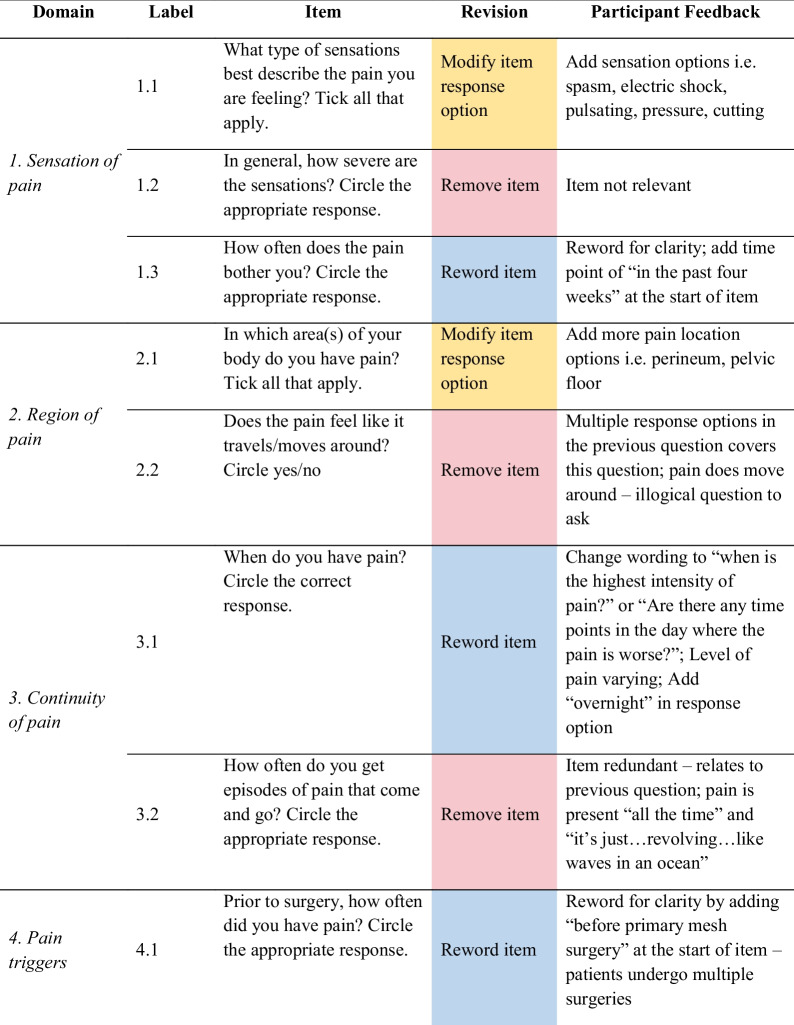

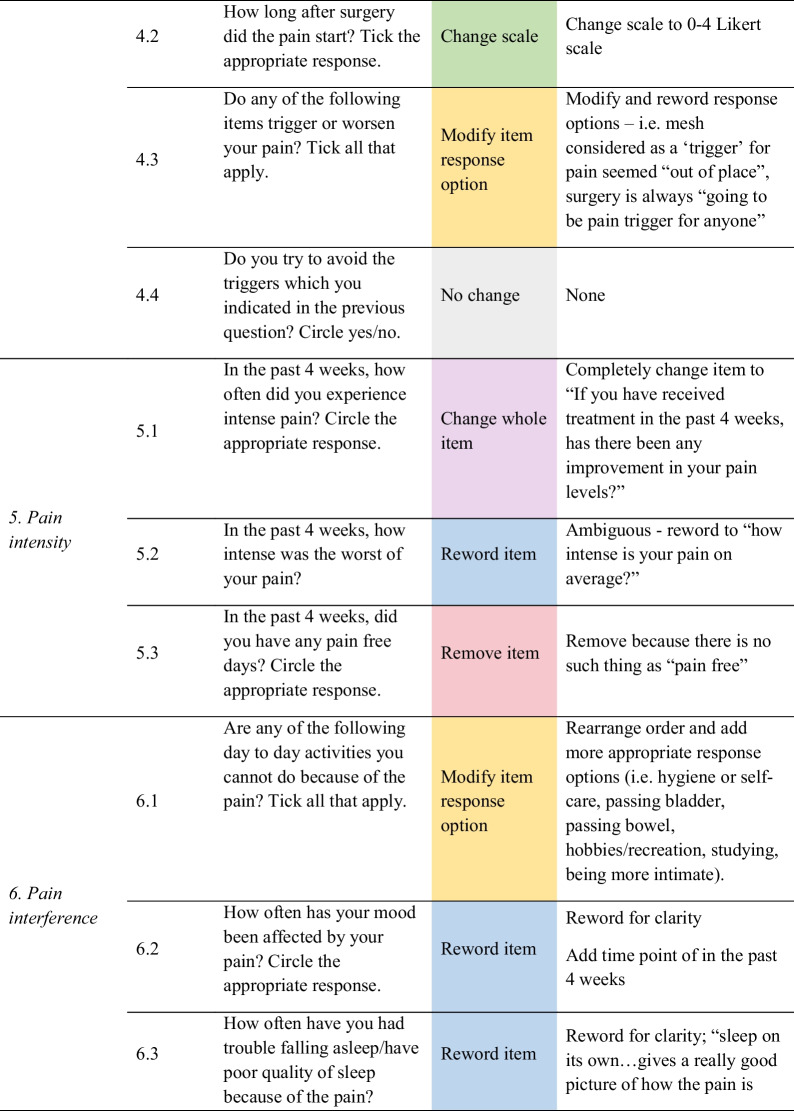

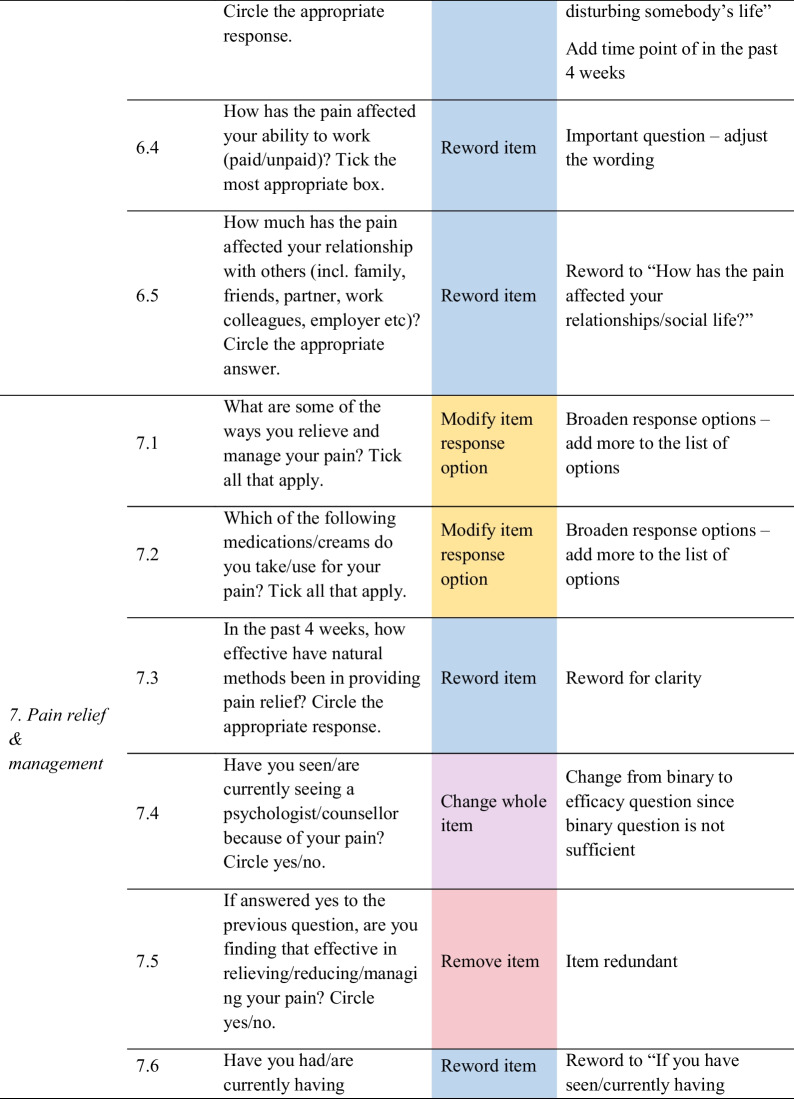

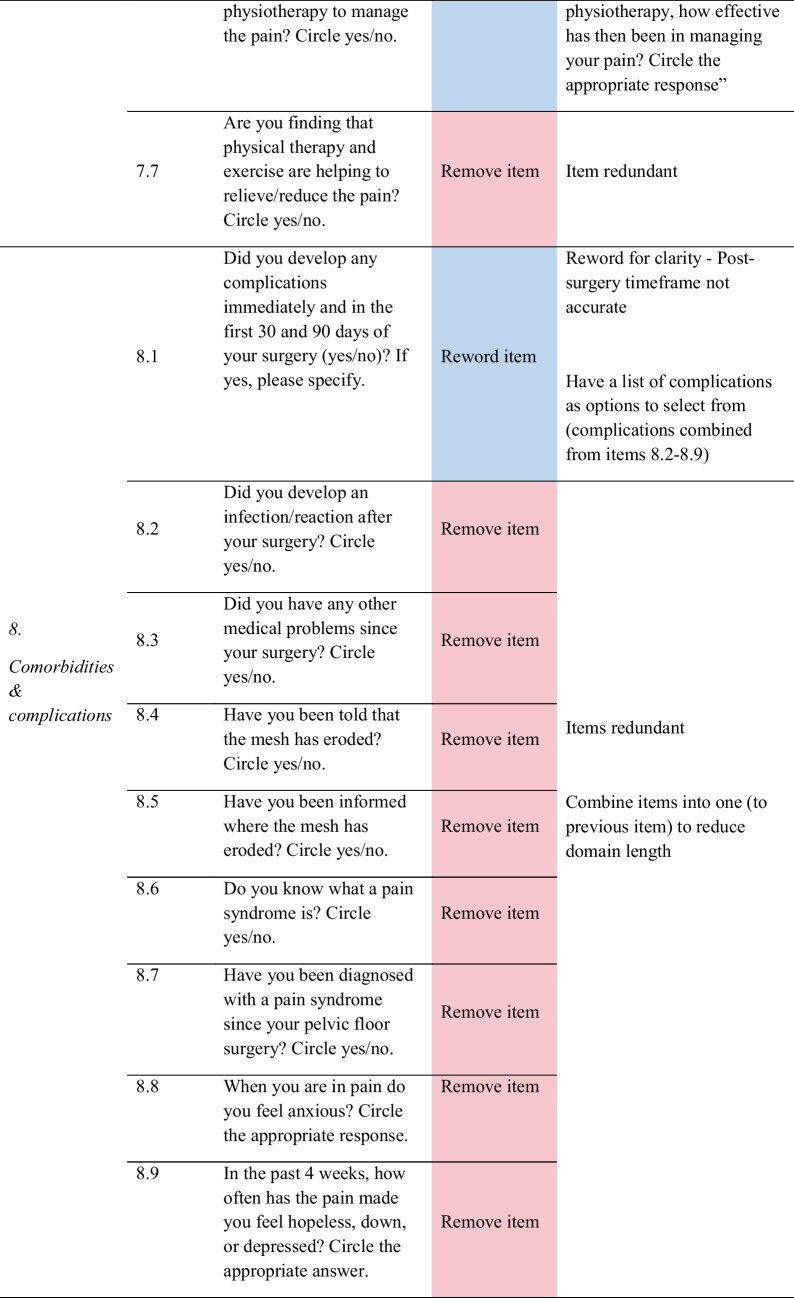
Item refinement table

#### Feedback from Women for Items in Each Domain

The key feedback for items in *domain 1 – sensation of pain* included adding additional pain sensations (i.e. spasm, electric shock, pulsating and cutting); removing reference to pain severity; and pre-empting questions regarding pain in the past 4 weeks (Table 3). Most women typically experience chronic pain post-pelvic floor surgery that is present ‘all the time… just the intensity that varies’. For items relating to *domain 2 – region of pain,* women suggested including more pain locations (i.e. perineum, pelvic floor, stomach/whole abdomen, bladder, bowel); and removing reference to whether pain moves around as it was not logical to ask as it is typical and assumed for the pain to move around among women.

In *domain 3 – continuity of pain*, the main feedback from women was reword the question referring to when pain occurs because women may ‘have [pain] all the time, but…the levels vary’; and remove reference to frequency of intermittent pain as it was related to previous item, where the pain is present “all the time” and ‘it’s just…revolving…like waves in an ocean’ (Table 3). Feedback for *domain 4 – pain triggers* included rewording item 4.1 to specify which surgery was referred to as patients often undergo multiple surgeries; and changing the scale in item 4.2 to a 0–4 Likert scale; modify and reword response options in item 4.3 as they were ‘out of place’ and not relevant (i.e. surgery is always ‘going to be pain trigger for anyone’). There was no feedback for item 4.4. This was the only item participants agreed to keep as is out of the 35 items.

All three items in *domain 5 – pain intensity* had to be refined. Women suggested changing an item completely, rewording an ambiguous question, and removing reference to pain-free days. Feedback was provided for *domain 6 – pain interference* to reorder response options and add more appropriate options (i.e. hygiene or self-care, passing bladder, passing bowel, etc.) but ensuring that the item is not ‘too long’ or ‘people will give up’. Women recommended rewording the four remaining items for clarity, and adding the time point of in the past 4 weeks for items 6.2 and 6.3 (Table 3).

For *domain 7 – pain relief and management*, feedback comprised adding more response options for items associated with ways to relieve and manage, and the types of medications taken or creams used for the pain. There was feedback to reword two of the items on the effectiveness of natural methods and physiotherapy on pain for clarity, changing a whole item about seeing a psychologist/counsellor from a binary to efficacy question, and removing two redundant items.

*Domain 8 – comorbidities and complications* had the highest number of items that women wanted removed to reduce domain length, and some items were redundant. Women suggested combining the removed items as response options of complications into the first item of the domain (item 8.1), which asks about the complications developed post-surgery. There was also a recommendation to include additional complications in the item (i.e. nerve damage, mental health concerns, organ damage/failure). Overall, women found this domain important as it examined other problems experienced post-pelvic floor surgery alongside chronic pain.

### Preliminary Version of Pain-Specific PROM

The first draft (preliminary version) of the instrument was developed on the basis of feedback from women through focus groups and interviews, and from APFPR Steering Committee clinicians through structured discussions (see Supplementary Material 4). Following these discussions with clinicians, five items were removed and response options were revised to ensure consistency and applicability. The preliminary version of the PROM consists of 16 items under seven domains (1) region of pain, (2) pain triggers, (3) sensation of pain, (4) intensity and continuity of pain, (5) pain interference, (6) comorbidities and complications and (7) pain relief and management. In this version, the domains have been reordered, and the domains of pain intensity and continuity of pain have been combined into one. This PROM is designed to be broadly applicable to include patients following a pelvic floor procedure, including those who undergo native tissue repair.

## Discussion

This was a qualitative study engaging women from the APFPR to pretest and select items for inclusion in a new pain-specific measure for women with PFDs post-surgery. Feedback was also obtained through structured discussions with clinicians from the APFPR Steering Committee. The pretesting through focus groups/interviews helped to identify any issues with initial content and formatting of items, guide decision-making regarding changes to the items and shape the design of the instrument before formal psychometric evaluation [15, 18]. Women participating in the discussions demonstrated a deep understanding of their pain, which assisted in informing a more accurate reflection of their experiences, despite the uncomfortable nature of the questions.

The focus groups/interviews revealed that not all concepts were relevant to the target population. The largest number of items was removed from the domain of comorbidities and complications, as women perceived this domain was too lengthy. However, for this domain, several adverse events that women experienced were recommended to be added, including mental health concerns. One study found that women with the most postoperative PFD symptoms were more likely to have depression and anxiety symptoms after pelvic floor surgery [19]. Another study demonstrated that women experiencing mesh-related complications were psychologically traumatised, with high levels of anxiety and fear, and had suicidal thoughts [20].

In this study, the wording and response options of the items were also revised for clarity and comprehension. Women provided alternative suggestions in rewording and changing the items that they felt were suitable for this cohort based on their knowledge and lived experience of PFD and pelvic floor surgical pain. For example, participants perceived a four-point response scale as more appropriate for the candidate item assessing the timing of postoperative pain onset, which was originally presented using single-option tick boxes. However, likely, participants did not interpret the term ‘Likert scale’ as it is understood in psychometrics.

Some items in the draft list may have appeared similar; however, they were designed to assess distinct constructs. For instance, one item focused on the frequency and bother of pain, while another captured the timing of pain onset. Each item received individual feedback during focus group discussions, and the aim was to reduce redundancy and enhance the distinctiveness of the PROM. In several cases, participants recommended rewording one item while suggesting the removal of another with similar wording. To address this, items were further refined to ensure conceptual clarity, eliminate redundancy and improve relevance. This iterative process of item revision ensured that the final PROM did not contain duplicate or overlapping items.

The focus groups/interviews emphasised the importance of how item response options are ordered and presented in a more meaningful way for women in the instrument design. Overall, from the pretesting, all items except one underwent revision and refinement to be acceptable and appropriate for women with PFDs.

Prior research was conducted to pretest an instrument or items for a new instrument using cognitive interviews or focus groups, but not specific to pain relating to PFD and pelvic floor surgery [19–22]. Despite this, similar to the findings of this study, previous studies showed that pretesting through focus groups identified general usability issues relating to the wider application or implementation of the specific measure in clinical practice [19–22].

One of the main strengths of this study was being able to gather detailed insight and receive diverse, valuable feedback and context from women with lived experience of pain following PFD surgery and from clinicians on items to include in the new pain instrument [23]. Study limitations included a lack of randomisation as participants were not selected randomly, hence potentially limiting the generalisability of results [24, 25]. Our study also had a small sample size, unequal distribution of participants across the groups, and biased responses from women (indication of self-report bias/measurement error). Recall bias may also be present, especially when patients responded to questions regarding their surgery and PFD diagnosis in the demographic questionnaire.

In addition, although the PROM has been designed for women experiencing pain following any pelvic floor procedure (as reflected in the first draft of the instrument), most participants were recruited through online support groups and consumer channels predominantly involving women with mesh-related experiences. Therefore, the study sample did not include women who underwent native tissue repair alone. This limitation may affect the generalisability of the findings to that subgroup [26].

## Conclusion

This study involved using focus groups to test items for inclusion in a new PROM for pain in women post-PFD surgery. Pretesting of items is an important step in the development of a new instrument. Our study highlighted key issues with the content, structure and relevance of the items. Through this, we were able to revise the items and produce the preliminary version of the pain measure for pelvic floor procedure. The instrument developed has been informed by key stakeholders; however, it has not yet been sufficiently refined or validated for use. The next steps involve the psychometric evaluation of the target population (including mesh and non-mesh cases), which will provide a rationale for the further reduction of items/responses, confirm the structure of the PROM and provide initial essential evidence of PROM data quality, reliability and validity. Once the new PROM is fully developed and validated, it is hoped that it will monitor health outcomes and better identify and enable patient–clinician discussion of existing unmet needs of women with chronic pain following pelvic floor surgery in clinical care settings (i.e. hospitals, specialist visits).

## Supplementary Information

Below is the link to the electronic supplementary material.

Supplementary file1 (DOCX 36 KB)

Supplementary file2 (DOCX 28 KB)

Supplementary file3 (DOCX 23 KB)

Supplementary file4 (DOCX 46 KB)

## Data Availability

Data sharing does not apply to this article as no datasets were generated and/or analysed during the current study.
