# Supplementary material for: Patient and Clinician Feedback to Inform the Development of a New Pain-Specific Patient-Reported Outcome Measure for Pelvic Floor Surgery
Source: Int Urogynecol J. 2025 Aug 1;36(12):2473–83. doi: 10.1007/s00192-025-06248-1 (PMC12756322; doi:10.1007/s00192-025-06248-1)
Supplement: Supplementary file 1 — Supplementary file1 (DOCX 36 KB) [file 192_2025_6248_MOESM1_ESM.docx]

**Supplementary Material 1:** Proposed list of items presented to focus groups (n=35)

| **Item number** | **Domain** | **Subdomain** | **Restated/refined item** | **Scale** | **Reason for choosing scale** |
| --- | --- | --- | --- | --- | --- |
| 1.1 | 1.Sensation of pain | 1. Sharp, stabbing, Shooting 2. aching, throbbing, dull 3. Numbness, cold 4. Pulling, dragging, ripping 5. Burning 6. Cramping, spasm | What type of sensations best describe the pain you are feeling? Tick all that apply. | - sharp - stabbing - poking - shooting - aching - cramping - throbbing - dull - numbness - tingling - pulling - dragging - ripping - burning - stinging | Allow respondents to select multiple answers from the list of choices |
| 1.2 | 1.Sensation of pain | 1. Sharp, stabbing, Shooting 2. aching, throbbing, dull 3. Numbness, cold 4. Pulling, dragging, ripping 5. Burning 6. Cramping, spasm | In general, how severe are the sensations? | Likert scale: 0=None/no sensations 1=Mild  2=Moderate  3=Severe 4=Very severe | Severity question |
| 1.3 | 1.Sensation of pain | 1. Sharp, stabbing, Shooting 2. aching, throbbing, dull 3. Numbness, cold 4. Pulling, dragging, ripping 5. Burning 6. Cramping, spasm | How often does the pain bother you? Circle the appropriate response. | 0=None of the time  1=A little of the time  2=Some of the time  3=Most of the time 4=All of the time | Bother/frequency question |
| 2.1 | 2.Region of pain | 1. Leg, thigh and toe 2. Groin 3. Abdomen 4. Back and spine 5. Buttock 6. Genitals 7. Pelvis and hip 8. Upper body | In which area(s) of your body do you have pain? Tick all that apply. | - leg, - thigh - calf - and - toe - groin - lower abdomen - back and spine - buttock - genitals (vulva, vagina) - pelvis, - pubic bone - hip - upper body | Allow respondents to select multiple answers from the list of choices |
| 2.2 | 2.Region of pain | 1. Leg, thigh and toe 2. Groin 3. Abdomen 4. Back and spine 5. Buttock 6. Genitals 7. Pelvis and hip 8. Upper body | Does the pain feel like it travels/moves around? Circle yes/no. | 0=No  1=Yes | Binary question |
| 3.1 | 3.Continuity of pain | 1. Constant 2. Intermittent | When do you have pain? Circle the appropriate response. | 0=Not sure  1=In the morning  2=In the evening  3=At night  4=24 hours a day | Measure time points to when pain occurs |
| 3.2 | 3.Continuity of pain | Intermittent | How often do you get episodes of pain that come and go? Circle the appropriate response. | 0=None of the time  1=A little of the time  2=Some of the time  3=Most of the time 4=All of the time | Frequency question |
| 4.1 | 4.Pain triggers | Surgery | Before surgery, how often did you have pain? Circle the appropriate response. | 0=None of the time/did not have pain  1=A little of the time  2=Some of the time  3=Most of the time 4=All of the time | Frequency question |
| 4.2 | 4. Pain triggers | Surgery | How long after surgery did the pain start? Tick the appropriate response. | - immediately after, when woke up - 24 hours - 48 hours - 72 hours - 1 week after - 2 weeks after - 3 weeks after - 6 months after - 1 year after | Single option tick box – allow respondents to select out of the many choices |
| 4.3 | 4. Pain triggers | 1. Surgery 2. Intercourse 3. Heightened emotions 4. Bladder and bowel 5. Mesh 6. Food and drink 7. Activities, movement | Do any of the following items trigger or worsen your pain? Tick all that apply. | - intercourse - stress, anxiety - heightened emotions - physical activity/exercise - normal walking - lying down - sitting - lifting - standing - sugary food - processed food - salty food - caffeinated drinks - alcohol - juice/cordial - soft drinks - passing bladder or bowels - pelvic mesh | Allow respondents to select multiple answers from the list of choices |
| 4.4 | 4. Pain triggers | 1. Surgery 2. Intercourse 3. Heightened emotions 4. Bladder and bowel 5. Mesh 6. Food and drink 7. Activities, movement | Do you try to avoid the triggers which you indicated in the previous question? Circle yes/no. | 0=No  1=Yes | Binary question |
| 5.1 | 5. Pain intensity | Covers mild and severe pain but may also cover all subdomains (none, mild, moderate, severe) | In the past 4 weeks, how often did you experience intense pain? Circle the appropriate response. | 0=never  1=rarely  2=sometimes 3=often 4=always | Frequency question |
| 5.2 | 5. Pain intensity | 1. None 2. Mild 3. Moderate 4. Severe | In the past 4 weeks, how intense was the worst of your pain? Circle the appropriate response. | 0=None/no sensations 1=Mild  2=Moderate  3=Severe 4=Very severe | Intensity/severity question |
| 5.3 | 5. Pain intensity | None | In the past 4 weeks, did you have any pain free days? Circle yes/no. | 0=No  1=Yes | Binary question |
| 6.1 | 6. Pain interference | Activities, movement | Are any of the following day-to-day activities you cannot do because of the pain? Tick all that apply. | - walking - sitting - physical activity/exercise - standing - daily chores (incl. cooking, cleaning, laundry...) - running errands - bathing/showering - looking after family - attending work - having sex - studying - attending university - catching up with friends/family | Allow respondents to select multiple answers from the list of choices |
| 6.2 | 6. Pain interference | Mood | How often has your mood been affected by your pain? Circle the appropriate response. | 0=None of the time  1=A little of the time  2=Some of the time  3=Most of the time 4=All of the time | Frequency question |
| 6.3 | 6. Pain interference | Sleep | How often have you had trouble falling asleep/have poor quality of sleep because of the pain? Circle the appropriate response. | 0=None of the time  1=A little of the time  2=Some of the time  3=Most of the time 4=All of the time | Frequency question |
| 6.4 | 6. Pain interference | Work | How has the pain affected your ability to work (paid/unpaid)? Tick the most appropriate box. | - None – able to work at full capacity - Had to reduce the hours I work - Had to reduce the number of days I work (move to casual/part-time) - Had to change career - Had to stop working completely | Tick box - allows respondents to select multiple answers from the list of choices |
| 6.5 | 6. Pain interference | Relation with other | How much has the pain affected your relationship with others (incl. family, friends, partner, work colleagues, employer etc)? Circle the appropriate response. | 0=Not at all  1=A little  2=Somewhat  3=Moderately  4=A great deal | Bother question |
| 7.1 | 7. Pain relief and management | 1. Electric current machines 2. Medication 3. Meditation 4. Natural remedies 5. Physical therapy and exercise 6. Psychological therapy | What are some of the ways you relieve and manage your pain? Tick all that apply. | - medication - natural remedies - massage/physio - psychological therapy - electric current machines - meditation | Allow respondents to select multiple answers from the list of choices |
| 7.2 | 7. Pain relief and management | Medication | Which of the following medications/creams do you take/use for your pain? Tick all that apply. | - paracetamol - non-steroidal anti-inflammatory drugs (nurofen, ibuprofen, advil…) - nerve pain medications/antidepressants (amitriptyline/endep, duloxetine, pregabalin/gabapentin) - narcotic (opioid) medications (codeine, oxycodone, tapentadol - anti-inflammatory suppositories - compounded cream - steroid ointment - Other medications not listed (please state):_____________ | Allow respondents to select multiple answers from the list of choices |
| 7.3 | 7. Pain relief and management | Natural remedies | In the past 4 weeks, how effective have natural methods been in providing pain relief? Circle the appropriate response. | 0=not at all effective 1=slightly effective  2=moderately effective 3=very effective 4=extremely effective | Efficacy question |
| 7.4 | 7. Pain relief and management | Psychological therapy | Have you seen/are currently seeing a psychologist/counsellor because of your pain? Circle yes/no. | 0=No  1=Yes | Binary question |
| 7.5 | 7. Pain relief and management | Psychological therapy | If answered yes to the previous question, are you finding that effective in relieving/reducing/managing your pain? Circle yes/no. | 0=No  1=Yes | Binary question |
| 7.6 | 7. Pain relief and management | Physical therapy & exercise | Have you had/are currently having physiotherapy to manage the pain? Circle yes/no. | 0=No  1=Yes | Binary question |
| 7.7 | 7. Pain relief and management | Physical therapy & exercise | Are you finding that physical therapy and exercise are helping to relieve/reduce the pain? Circle yes/no. | 0=No  1=Yes | Binary question |
| 8.1 | 8. Comorbidities and complications | 1. Mesh exposure 2. Pain syndromes 3. Other disorders 4. Infection or reaction 5. Mental health disorders | Did you develop any complication(s) immediately and in the first 30 and 90 days of your surgery (yes/no)? If yes, please specify. | 0=No  1=Yes | Binary question |
| 8.2 | 8. Comorbidities and complications | Infection or reaction | Did you develop an infection or reaction after your surgery? Circle yes/no. | 0=No  1=Yes | Binary question |
| 8.3 | 8. Comorbidities and complications | Other disorders | Did you have any other medical problems since your surgery? Circle yes/no. | 0=No  1=Yes | Binary question |
| 8.3 | 8. Comorbidities and complications | Mesh exposure | Have you been told that the mesh has eroded? Circle yes/no. | 0=No  1=Yes | Binary question |
| 8.4 | 8. Comorbidities and complications | Mesh exposure | Have you been informed where the mesh has eroded? Circle yes/no. | 0=No  1=Yes | Binary question |
| 8.5 | 8. Comorbidities and complications | Pain syndrome | Do you know what a pain syndrome is? Circle yes/no. | 0=No  1=Yes | Binary question |
| 8.6 | 8. Comorbidities and complications | Pain syndrome | Have you been diagnosed with a pain syndrome since your pelvic floor surgery? Circle yes/no. | 0=No  1=Yes | Binary question |
| 8.7 | 8. Comorbidities and complications | Mental health disorders | When you are in pain do you feel anxious? Circle yes/no | 0=No  1=Yes | Binary question |
| 8.8 | 8. Comorbidities and complications | Mental health disorders | In the past 4 weeks, how often has the pain made you feel hopeless, down or depressed? Circle the appropriate response. | 0=none of the time 1=rarely 2=some of the time 3=most of the time 4=all the time | Frequency question |
