# Supplementary material for: Patient and Clinician Feedback to Inform the Development of a New Pain-Specific Patient-Reported Outcome Measure for Pelvic Floor Surgery
Source: Int Urogynecol J. 2025 Aug 1;36(12):2473–83. doi: 10.1007/s00192-025-06248-1 (PMC12756322; doi:10.1007/s00192-025-06248-1)
Supplement: Supplementary file 2 — Supplementary file2 (DOCX 28 KB) [file 192_2025_6248_MOESM2_ESM.docx]

**Supplementary Material 2:** Focus Group Discussion Guide

WELCOME AND GROUND RULES

- Share welcome slide

Thank you for agreeing to be part of this focus group. We appreciate your willingness to participate. Before we have introductions, I’d like to provide some background information to set the stage for your conversation.

**Introduction**

*Purpose of focus groups:* We are conducting a few focus groups as part of PhD project on the development of a new pain-specific questionnaire for women with pelvic floor disorders. We are having these focus groups because we want to gain your input and feedback on the wording and scaling of each proposed question for the new questionnaire. We would you to provide solid ideas on how the questions should be formulated in our discussions.

*Informed consent:* Each person has been provided with an information sheet. By being in this meeting, you agree to participate in this focus group. You may choose not to answer a question if you would prefer not to. You may also stop your participation at any time.

We will be recording our discussion today to accurately capture all your comments. To protect your privacy, names will not be connected to what you say and only our project team will have access to the recordings. We will take notes on what was said, not who said what.

Please confirm that it is OK to audio-record this meeting. You may do so verbally by saying “Yes” or putting your response in the chat box.

[CLICK RECORD ONCE EVERYONE CONFIRMS]

Ground rules

- Provide slide with ground rules

Today’s discussion will last up to an hour (60 minutes)

1. WE WANT YOU TO DO THE TALKING.

- We would like to hear from everyone
- Let’s make sure one person speaks at a time for us to go back to the recording and hear what everyone said.
- I may call on you if I have not heard from you in a while.
- If any questions or thoughts come to mind as others are speaking, feel free to add them to the chat.

2. THERE ARE NO RIGHT OR WRONG ANSWERS

- We want your honest opinions and reactions.
- Every person’s experience and opinion are important.
- Speak up whether you agree or disagree.
- We want to hear a wide range of opinions.
- We should be respectful of one another even when we disagree.

3. WHAT IS SAID IN THIS ROOM STAYS HERE

- We want everyone to feel comfortable and safe sharing as this will help us to get the best information

4. WE WILL BE RECORDING THE DISCUSSION

- We want to capture everything you have to say.
- We do not identify anyone by name in our report and you will remain anonymous.
- Speak loudly so we can hear you and what you say in the recording is clear.
- Please limit background noise by muting yourself when we or other participants are talking.

Key information

- Provide slide with key information

- Please note that we want to have fewer items but have a decent scale for the new pain questionnaire.
- We also do not want to generate/include open-ended questions

We have a lot to talk about today, so there may be times when I need to move the discussion along. Please understand that when we ask that we move to a new topic or speaker, we do not mean to be rude, we just want to be very considerate of your time.

Any questions before we get started?

Please show a thumbs up reaction.

Great, let’s get started.

Thank you everyone and nice to virtually meet all of you. We greatly appreciate your participation.

DISCUSSION ON THE PROPOSED LIST OF ITEMS AND SCALING

Go through each item and the scaling ask participants to provide comments/feedback or if there is anything they want to change. If there are any new proposed items, ask participants to add them to the chat.

*Key question (for each item):*

- What is your take/thoughts on this item?

- Is this item appropriate/relevant to include in the final questionnaire?

Probes:

- What is about this item that you prefer/like/dislike?

- Can you share your reasons for that decision?

- How could you improve this item?

- Can you show/demonstrate what you mean?

- Is there anything else to add?

CLOSING

- Share concluding slide

We really appreciate speaking with you all today and have learned a lot. We are almost done with our discussion. Before we all leave:

- Is there anything else that you would like to add about any of the topics that we’ve discussed?
- Is there anything else that you think is important for me to know about your experiences with pain from PFDs and associated surgery?
- Do you have any questions for me before we say goodbye?

Thank you for your time and participation in this discussion. Hope you have a great rest of your day.
