# Supplementary material for: Patient and Clinician Feedback to Inform the Development of a New Pain-Specific Patient-Reported Outcome Measure for Pelvic Floor Surgery
Source: Int Urogynecol J. 2025 Aug 1;36(12):2473–83. doi: 10.1007/s00192-025-06248-1 (PMC12756322; doi:10.1007/s00192-025-06248-1)
Supplement: Supplementary file 3 — Supplementary file3 (DOCX 23 KB) [file 192_2025_6248_MOESM3_ESM.docx]

**Supplementary Material 3:** Demographic Questionnaire

*Please fill in the blanks or place an X or ✓ next to the word/phrase that best matchers your response.*

Full name:______________ Date:______________

1. DOB:___­­­­­___________ (dd/mm/yyyy)
2. What is your current marital/relationship status?

|  | Married |
| --- | --- |
|  | Single |
|  | Divorced |
|  | Separated |
|  | Widowed |
|  | Living with partner |
|  | Engaged |
|  | Prefer not to say |

1. What is the highest degree or level of school you have completed?

|  | Year 11 or below (includes Certificate I/II/not further defined) | |
| --- | --- | --- |
|  | Year 12 |  |
|  | Certificate III/IV |  |
|  | Advanced Diploma and Diploma |  |
|  | Bachelor Degree |  |
|  | Graduate Diploma and Graduate Certificate |  |
|  | Postgraduate Degree |  |

1. What kind of pelvic floor disorder do you have?

|  | Stress urinary incontinence | |
| --- | --- | --- |
|  | Pelvic organ prolapse |  |
|  | Both incontinence and prolapse |  |

|  |
| --- |

1. When were you diagnosed with a pelvic floor disorder? (Please provide the year)

|  |
| --- |

1. When did you have pelvic floor surgery?

(Please provide the year)

1. What type of pelvic floor surgery did you have?

|  |
| --- |

1. Are you aware if mesh implant was used as part of your repair?

|  | Yes | |
| --- | --- | --- |
|  | No |  |
|  | Unsure |  |

1. Did you experience/are you experiencing pain relating to your pelvic floor disorder and/or surgery?

|  | Yes | |
| --- | --- | --- |
|  | No |  |
|  | Unsure |  |

1. When did you start experiencing the pain? (Please provide the year)

|  |
| --- |
