# Supplementary material for: Patient and Clinician Feedback to Inform the Development of a New Pain-Specific Patient-Reported Outcome Measure for Pelvic Floor Surgery
Source: Int Urogynecol J. 2025 Aug 1;36(12):2473–83. doi: 10.1007/s00192-025-06248-1 (PMC12756322; doi:10.1007/s00192-025-06248-1)
Supplement: Supplementary file 4 — Supplementary file4 (DOCX 46 KB) [file 192_2025_6248_MOESM4_ESM.docx]

**Supplementary Material 4:** Preliminary version of pain-specific PROM

**POST-PELVIC FLOOR PROCEDURE PAIN QUESTIONNAIRE (PPPQ)**

The Post-Pelvic Floor Procedure Pain Questionnaire (PPPQ) is a pain-specific instrument developed for women following their pelvic floor procedure. Before starting the questionnaire, please answer the brief list of demographic/screening questions. When completing the questionnaire, feel free to provide any feedback/suggestions in the comment box under each question to help us improve it.

| **Today’s date** *(dd/mm/yyyy)***: ___**/___/_____ |
| --- |
| 1. What is your age (years)? ____________ 2. Please provide the date of your recent pelvic floor procedure *(dd/mm/yyyy)*: **___**/___/_____ 3. Did you develop pain following your pelvic floor procedure?  - YES –  1. How soon after your procedure did you develop pain? *Please tick one option.*  \|  \| Within 1 week \| \| --- \| --- \| \|  \| Within 1 month \| \|  \| Within 6 months \| \|  \| Within 1 year \| \|  \| After more than 1 year \|  1. Please provide the date of your pelvic floor procedure to when the pain first started *(dd/mm/yyyy)*: / /  - Please answer ALL questions of the questionnaire below. - If you do not have pain, please answer questions 6 to 14 of the questionnaire. |

| **DOMAIN 1: REGION OF PAIN** |
| --- |
| **Q1. In which area(s) of your body do you experience pain?** *Tick all the apply.*   - 1 = Upper leg (e.g. thigh) - 1 = Buttock - 1 = Genitals (vulva, vagina) - 1 = Pelvis - 1 = Pubic bone - 1 = Hip - 1 = Bladder - 1 = Bowel - 1 = Skin/tissue - 1 = Lower leg (e.g. calf) - 1 = Foot - 1 = Groin - 1 = Upper torso - 1 = Upper arm and shoulder - 1 = Forearm - 1 = Lower abdomen - 1 = Back and spine |
| ***Please provide any feedback/suggestions you have to improve Q1.*** |

| **DOMAIN 2: PAIN TRIGGERS** |
| --- |
| **Q2. Please select items that trigger/worsen your pain.** *Tick all that apply.*   - 1 = Full bowel - 1 = Urinating - 1 = Having a bowel movement - 1 = Temperature - 1 = Intercourse - 1 = Orgasm - 1 = Menstrual cramps - 1 = Other not listed here - 1 = Stress/Anxiety - 1 = Heightened emotions - 1 = Trauma (physical, psychological) - 1 = Activity/Exercise (i.e. movement, physical activity) - 1 = Cessation of clinical exercise/therapy - 1 = Posture (lying down, sitting, standing) - 1 = Food and beverages - 1 = Full bladder |
| ***Please provide any feedback/suggestions you have to improve Q2.*** |
| **Q3. How often do you try to avoid the triggers you indicated in question 2?** *Tick* ***one*** *box only.*   - 0 = Never - 1 = Rarely - 2 = Sometimes - 3 = Often - 4 = Always |
| ***Please provide any feedback/suggestions you have to improve Q3.*** |

| **DOMAIN 3: SENSATION OF PAIN** |
| --- |
| **Q4. What type of sensation(s) best describes your pain?** *Tick all that apply.*   - 1 = Sharp - 1 = Dragging - 1 = Pulling - 1 = Ripping - 1 = Cutting - 1 = Pressure - 1 = Burning/stinging - 1 = Numbness - 1 = Tingling - 1 = Electric shock - 1 = Stabbing - 1 = Poking - 1 = Aching - 1 = Cramping - 1 = Spasm - 1 = Throbbing - 1 = Pulsating - 1 = Dull |
| ***Please provide any feedback/suggestions you have to improve question 4.*** |

| **DOMAIN 4: INTENSITY AND CONTINUITY OF PAIN** |
| --- |
| **Q5. On the days you have pain, when does it usually occur?** *Tick* ***one*** *box only.*   - In the morning - In the evening - Night time - Overnight - 24 hours a day (All the time)   *(This question is not scored)* |
| ***Please provide any feedback/suggestions you have to improve question 5.*** |
| **Q6. In the past 4 weeks, how intense was the worst of your pain?** *Tick* ***one*** *box only.*   - 0 = Not intense (did not have pain/sensations) - 1 = Mild - 2 = Moderate - 3 = Severe - 4 = Very severe |
| ***Please provide any feedback/suggestions you have to improve question 6.*** |
| **Q7. In the past 4 weeks, how often did you have pain-free days?** *Tick* ***one*** *box only.*   - 0 = Never - 1 = Rarely - 2 = Sometimes - 3 = Often - 4 = Always |
| ***Please provide any feedback/suggestions you have to improve question 7.*** |

| **DOMAIN 5: PAIN INTERFERENCE** |
| --- |
| **Q8. Has your pain interfered with any of the following activities/tasks?** *Tick all that apply.*   - 1 = Driving - 1 = Work - 1 = Studying - 1 = Volunteering - 1 = Sleeping - 1 = Being intimate - 1 = Urinating - 1 = Having a bowel movement - 1 = Bathing/showering - 1 = Hygiene/self-care - 0 = None - 1 = Daily chores - 1 = Looking after family - 1 = Walking - 1 = Posture (sitting, standing) - 1 = Physical activity, sport, exercise - 1 = Hobbies and leisure - 1 = Socialising - 1 = Attending medical appointments - 1 = Travel |
| ***Please provide any feedback/suggestions you have to improve question 8.*** |
| **Q9. In the past 4 weeks, how often has your mood been affected by pain?** *Tick* ***one*** *box only.*   - 0 = None of the time - 1 = A little of the time - 2 = Some of the time - 3 = Most of the time - 4 = All of the time |
| ***Please provide any feedback/suggestions you have to improve question 9.*** |
| **Q10. In the past 4 weeks, how often have you had trouble sleeping/had poor sleep because of pain?** *Tick* ***one*** *box only.*   - 0 = None of the time - 1 = A little of the time - 2 = Some of the time - 3 = Most of the time - 4 = All of the time |
| ***Please provide any feedback/suggestions you have to improve question 10.*** |
| **Q11. How does the pain impact your ability to work (paid/unpaid)?** *Tick* ***one*** *box only.*   - 0 = None – able to work at full capacity - 1 = Had to reduce work hours - 2 = Had to reduce the number of days working (move to casual/part-time) - 3 = Had to change career - 4 = Had to stop working completely |
| ***Please provide any feedback/suggestions you have to improve question 11.*** |
| **Q12. How much has the pain affected your relationships/social life?** *Tick* ***one*** *box only.*   - 0 = Not at all - 1 = A little - 2 = Somewhat - 3 = Moderately - 4 = A great deal |
| ***Please provide any feedback/suggestions you have to improve question 12.*** |

| **DOMAIN 6: COMORBIDITIES AND COMPLICATIONS** |
| --- |
| **Q13. Apart from pain, did you develop any other complications after your recent pelvic floor procedure surgery?** *Tick all that apply.*   - 1 = Organ damage/failure - 1 = Mental health/psychological problem(s) - 1 = Sleep disorder/lack of sleep - 1 = Phobias - 1 = Trauma/PTSD - 1 = Cognitive decline - 1 = Hypertension - 1 = Obesity - 0 = None ***– please go to Q15*** - 1 = Mesh exposure/erosion - 1 = Other pain disorders - 1 = Nerve damage - 1 = Infection/irritation/reaction - 1 = Inflammations - 1 = Autoimmune conditions - 1 = Loss of/reduced pelvic organ function - 1 = Recurrence of incontinence and/or prolapse |
| ***Please provide any feedback/suggestions you have to improve question 13.*** |
| **Q14. If you ticked ­at least one complication in Q13, how long after surgery did the complication(s) occur?** *Tick* ***one*** *box only.*   - 1 = Immediately after surgery - 2 = After one week - 3 = After one month - 4 = After one year - 5 = > 1 year after surgery |
| ***Please provide any feedback/suggestions you have to improve question 14.*** |

| **DOMAIN 7: PAIN RELIEF AND MANAGEMENT** |
| --- |
| **Q15. What are some of the ways you relieve and manage the pain??** *Tick all that apply.*   - 1 = Medications/creams/ointments - 1 = Natural remedies - 1 = Massage - 1 = Meditation - 1 = Physiotherapy - 1 = Psychological therapy - 1 = Acupuncture |
| ***Please provide any feedback/suggestions you have to improve question 15.*** |
| **Q16. If you ticked at least one option in Q15, how effective have they been in relieving and managing the pain?** *Tick* ***one*** *box only.*   - 1 = Extremely effective - 2 = Very effective - 3 = Moderately effective - 4 = Slightly effective - 5 = Not at all effective |
| ***Please provide any feedback/suggestions you have to improve question 16.*** |

**Thank you very much for answering these questions.**
